# Successful Multi-Level HPV Vaccination Intervention at a Rural Healthcare Center in the Era of COVID-19

**DOI:** 10.3389/fdgth.2021.719138

**Published:** 2021-08-19

**Authors:** Deanna Kepka, Kaila Christini, Emily McGough, Anna Wagner, Guilherme Del Fiol, Bryan Gibson, Shauna Ayres, Heather M. Brandt, Sara Mann, Amanda F. Petrik, Gloria D. Coronado

**Affiliations:** ^1^Cancer Control and Population Sciences, Huntsman Cancer Institute, University of Utah, Salt Lake City, UT, United States; ^2^College of Nursing, University of Utah, Salt Lake City, UT, United States; ^3^Telluride Regional Medical Center, Telluride, CO, United States; ^4^Department of Biomedical Informatics, University of Utah, Salt Lake City, UT, United States; ^5^St. Jude Children's Research Hospital and Comprehensive Cancer Center, Memphis, TN, United States; ^6^School of Medicine, University of Utah, Salt Lake City, UT, United States; ^7^Kaiser Permanente Center for Health Research, Portland, OR, United States

**Keywords:** HPV vaccination, rural, healthcare team training, text reminders, intervention–behavioral, visit reminder, patient education, missed opportunities

## Abstract

**Objectives:** To develop and test a human papillomavirus (HPV) vaccination intervention that includes healthcare team training activities and patient reminders to reduce missed opportunities and improves the rate of appointment scheduling for HPV vaccination in a rural medical clinic in the United States.

**Methods:** The multi-level and multi-component intervention included healthcare team training activities and the distribution of patient education materials along with technology-based patient HPV vaccination reminders for parents/caregivers and young adult patients. Missed vaccination opportunities were assessed pre- and post-intervention (*n* = 402 and *n* = 99, respectively) by retrospective chart review and compared using Pearson χ^2^. The patient parent/caregiver and young adult patient population (*n* = 80) was surveyed following the reminder messages and penalized logistic regression quantified unadjusted odds of scheduling a visit.

**Results:** Missed opportunities for HPV vaccination declined significantly from the pre-intervention to the post-intervention period (21.6 vs. 8.1%, respectively, *p* = 0.002). Participants who recalled receipt of a vaccination reminder had 7.0 (95% *CI* 2.4–22.8) times higher unadjusted odds of scheduling a visit compared with those who did not recall receiving a reminder. The unadjusted odds of confirming that they had scheduled or were intending to schedule a follow-up appointment to receive the HPV vaccine was 4.9 (95% *CI* 1.51–20.59) times greater among those who had not received the vaccine for themselves or for their child.

**Conclusions:** Results from this intervention are promising and suggest that vaccination interventions consisting of provider and support staff education and parent/caregiver and patient education materials, and reminders can reduce missed opportunities for vaccinations in rural settings.

## Background

Improving human papillomavirus (HPV) vaccine uptake could prevent tens of thousands of cancer cases each year. HPV is a sexually transmitted infection that causes cervical, anal, penile, vaginal, vulvar, and oropharyngeal cancers, and genital warts. While HPV vaccination can prevent most HPV-related cancers, more than 20,000 women and 14,000 men are diagnosed with HPV-associated cancers each year in the United States (1). This is troubling given that HPV vaccination rates remain substantially lower than national targets (80% by 2030 for adolescents aged 13–15) (2). Indeed, only about 54% of adolescents have met this target in more than 13 years after the vaccine was recommended for girls and 8 years after the recommendation for boys (3). Improving uptake of the HPV vaccine is a public health imperative.

Higher HPV vaccine uptake in rural communities could improve health outcomes for this unique population. The United States non-metropolitan residents have higher cervical cancer incidence, late-stage diagnoses, and death rates than metropolitan residents (4). Data from the 2019 National Immunization Survey-Teen show startlingly low HPV vaccine initiation and series completion rates for adolescents in rural regions. Rates of initiation or series completion are up to 10% points lower than for urban regions (5, 6). Vaccine registry data reveals that teens living in rural areas were 1.8 times more likely than urban residents to have a missed opportunity for HPV vaccination (when one receives another immunization and not the HPV vaccine) (7). *Developing and deploying effective strategies for improving rural HPV vaccine coverage is critical*.

Rural barriers to HPV vaccination are multi-factorial. A robust body of literature explicates barriers in receiving preventive health services, such as vaccination for HPV (8–11) and underscoring distinct challenges faced by rural residents (12, 13). At the patient level, individuals often lack awareness of the importance of vaccination, experience fear or fatalism, or are dissuaded by prevailing anti-vaccination norms (10, 14–17). Rural residents may experience limited healthcare access: studies show that rural adolescents are less likely than urban counterparts to attend a well-child visit and to receive a provider recommendation for HPV vaccination (6). In addition, providers and clinics are often limited by a lack of systematic methods for identifying patients eligible for vaccination; inadequate reimbursement and time for counseling about vaccination; and follow-up systems that do not track intervals for repeated doses (18). Rural clinics also face shortages of medical providers, especially pediatricians, well-versed in delivering adolescent vaccines (11, 19, 20).

Complicating the situation further, in March 2020, the WHO classified COVID-19 (i.e., coronavirus) as a global pandemic leading to an unprecedented strain on the U.S. healthcare system (21); nevertheless, it presents a teachable moment to communicate the importance of vaccination to counter anti-vaccination sentiment and improve rates of HPV vaccination. In the United States, the vast majority of non-essential medical care (e.g., well-child visits) did not occur during the peak of the COVID-19 pandemic (22, 23). In the early months of the pandemic, up to 40% of appointments for children's immunizations and 80% of appointments for teen's HPV vaccinations have been missed (24). As well-child visits have been resuming, adolescents have been the least likely to catch-up on immunizations, compared to younger children and infants (25). Furthermore, publicly-insured adolescents have experienced larger declines in immunization since the start of the pandemic in March 2020 than privately insured adolescents (3, 26).

Multi-level interventions are needed to reduce HPV vaccination disparities. Solving complex public health issues requires consideration of factors at multiple levels, such as the individual (e.g., fear, fatalism, lack of awareness, and misperception of threat), clinician (e.g., missed opportunities), clinic (e.g., limited operating hours), community, and society (e.g., low awareness and prioritization of vaccine). Current studies find that multi-level interventions display a synergistic effect, with interventions targeting parents and providers achieving higher levels of HPV vaccine uptake than interventions targeting each group alone. However, many of these past studies have not been rigorously tested in rural populations.

This study developed and tested a multi-level and multi-component intervention in a rural Telluride, Colorado, United States. The intervention consisted of healthcare team training activities to strengthen and increase strong and consistent HPV vaccination recommendations and evidence-based patient-directed HPV vaccine education materials as well as technology-based parent/caregiver reminders to make vaccine appointments for their age-eligible children. This study sought to fill important gaps in the literature, including exploring the impact of combining technology-based interventions with human-delivered communication. Few previous studies have tested the combination of strong provider recommendations for the vaccine with automated reminders to patients.

## Methods

### Study Location

Telluride Regional Medical Center (TMC) serves rural patients in the western United States. Telluride, Colorado is a rural community of <2,500 residents. In addition, Telluride is a mental health and primary care Health Professional Shortage Area (HPSA) and a Medically Underserved Area (MUA). Located in a box canyon of the Colorado Rocky Mountains in San Miguel County, Telluride is surrounded by mountains, receives over 167 inches of snowfall annually, and driving conditions are often treacherous. The nearest city to Telluride is Montrose (<20,000 people) and is approximately 1.5 h drive along over 65 miles of mountainous roads. TMC includes 5–8 primary care clinicians and approximately 7–10 other clinic staff members. They provide primary care services to all ages, from birth to death and are about 100 miles from the nearest hospital. The clinic uses eClinicalWorks^®^ (eCW) for the electronic health record (EHR). They have prioritized increasing HPV vaccination rates in 2017 when they began collaborating with Dr. Kepka, an associate professor at the University of Utah, United States and an investigator at Huntsman Cancer Institute, and the Intermountain West HPV Vaccination Coalition (led by Dr. Kepka) (27).

### Healthcare Team Training—Telluride Medical Center Provider Facilitation and Education Campaign

Dr. Kepka assembled a University team to guide the development and implementation of the components of the multi-level HPV vaccination intervention. Team members included two clinical informatics experts, a graduate student in nursing, a program manager and data analyst, and Dr. Kepka (an HPV vaccination expert and health services researcher). The development of the parent/caregiver and young adult reminder intervention included three video calls between the University team and TMC providers and support staff at the end of 2020. Calls consisted of an environmental scan of existing HPV vaccination efforts, a discussion on possible approaches for improving HPV vaccination rates and delivery, and conversations around the facilitation of TMC provider and support staff vaccination efforts by the University team. The video calls also included a walkthrough of the EHR workflow and immunization preparation activities at TMC. The team determined prioritization of the target population, the development of a vaccination patient reminder campaign and messaging content, and campaign implementation within the EHR. The team also completed a walkthrough of running the campaign with TMC team members as completed *via* virtual meeting. A step-by-step documentation and guide for EHR implementation developed by the University team were provided offline to the providers and support staff at TMC.

Human papillomavirus vaccination training for the healthcare team included two 1-h early morning video calls that focused on training providers and support staff at TMC on evidence-based HPV vaccination systems, vaccine recommendations, and patient education materials relevant to their patient population. Healthcare teams were taught how to deliver a strong, brief, and consistent provider recommendation for the HPV vaccine to their patients (28). They were also taught to treat every patient visit like a vaccination visit regardless of whether the child or young adult were at TMC for vaccinations. Last, healthcare team members were given evidence-based patient center HPV vaccination education materials (28). These training activities were facilitated by Dr. Kepka and the graduate student in nursing and were conducted in early 2021. Breakfast was provided to the TMC team as a thank you gesture for attending the healthcare team training activities.

Before and after the two training activities, an online survey that included HPV and HPV vaccination knowledge questions and barriers to vaccination was completed online by providers and support staff at TMC using Research Electronic Data Capture (REDCap) tools hosted at the University of Utah (29, 30). The pre-test survey was administered 2-weeks prior to the first training and the post-test survey was delivered about 2-weeks after the last training. Healthcare team survey respondents received an HPV vaccination coffee mug or lunch bag, a chance at a raffle prize after the first survey, and a $50 gift card after the completion of the second survey and training activities. Survey results, such as HPV vaccination barriers identified by providers and support staff, were examined. The percentage of providers and support staff selecting a given barrier was calculated from the total number of provider and support staff survey respondents. The percentage change described differences between pre- and post-intervention. Frequencies for provider and support staff self-report of feasibility and usability were obtained and compared using Pearson's χ^2^.

### Reminder Message Campaign

With the assistance of the University team, TMC providers and support staff developed a HPV vaccination reminder campaign for patients/caregivers with age-eligible children for the HPV vaccine (children ages 11–17) and young adults (ages 18–26) who are also age eligible for the HPV vaccine. The reminder message was branded as coming from TMC instead of sending reminders from their individual provider. HPV vaccination messages reminding patients or their parents/caregivers to schedule an appointment were sent from the Medical Center *via* patient preferred method (i.e., text or email) using patient outreach capabilities provided by the EHR system available at TMC (eClinicalWorks^®^).

Human papillomavirus vaccination reminder messages were designed using evidence-based recommendations highlighted by the American Cancer Society's HPV Vaccination Roundtable (28). First, the team pilot-tested a HPV vaccination reminder message directed at parents and caregivers of 11-year-olds to a small sample of parents/caregivers at TMC (*n* = 44) in August 2020. Then, the team improved the design of the reminder campaign and expanded the target group to parents and caregivers of children ages 11–17 who are age-eligible for the HPV vaccine and to young adults ages 18–26 who are also age-eligible for the vaccine. The final round of the HPV vaccination reminders was sent to the larger group of participants in October 2020.

An example reminder message that was sent *via* text or email to parents/caregivers at TMC during the reminder campaign is listed as:

*You'd do anything to protect your child*.
*Now is the time to give [NAME] the gift of cancer prevention*.
*The HPV vaccine protects boys and girls against up to 6 types of cancer*.
*Our records indicate that [NAME] has turned 11 since the beginning of the COVID-19 pandemic. We recommend TDaP, HPV, and Meningitis routine vaccines at the 11-year-old visit. Don't delay, get your child scheduled today for a well-child visit by calling (###) ###-####*.
*We are taking special precautions to ensure all well-child visits are safe during these challenging times*.

### Chart Review

A cross-sectional retrospective chart review of HPV immunization of all vaccine-eligible patients (ages 11–26 years) visiting the clinic prior to intervention (September 01 to December 01, 2019, *n* = 402) and a shorter period of time following the intervention (January 15 to March 15, 2021, *n* = 99) was performed. Age at the time of the visit was collected as a continuous variable and included in analyses as whole years (with no rounding). All other collected variables were binarized into yes/no categories and Pearson's χ^2^ with probabilities were calculated for pre- and post-intervention comparisons. To determine the robustness of χ^2^ estimates, sensitivity analyses were performed to examine the effects of partially overlapping samples. A dataset of non-overlapping individuals between pre- and post-interventions was created, and *p*-values compared for pre-intervention, post-intervention, and post-intervention without overlaps. Age distributions were compared using 95% confidence intervals for the median with the null-hypothesis locational parameter equal to the pre-intervention median. Analyses were performed using SAS software (version 9.4, SAS Institute, Cary, NC).

### Post-Intervention Parent/Caregiver and Young Adult Patient Survey

A short online survey was conducted among the TMC parent/caregiver and young adult patient population about 3 weeks following the vaccination reminder intervention. Participants received an email invitation to the survey with a URL link that was used to take the survey. The survey asked patients, parents, and/or caregivers if they had received a reminder; what mode (email, text, or telephone) of reminder they received; if they scheduled, or planned to schedule, an appointment; if they or their child had received the HPV vaccine; two open-ended qualitative response questions about scheduling the appointment and comments about the campaign; and demographic questions. Participants had the option to receive a $10 gift card as a thank you gift for their time upon completion of the survey. The survey was designed and administered using the online SurveyMonkey application (SurveyMonkey, Inc., San Mateo, California, USA, www.surveymonkey.com).

Survey data were analyzed descriptively using frequencies, distributions by scheduling (or intent to schedule) an appointment, and Pearson's χ^2^ with probabilities. Unadjusted odds ratios (cORs) were calculated for the odds of scheduling an appointment by age, gender, receiving a reminder, mode of reminder, and receipt of the vaccine for either self or child. Analyses were performed using SAS software (version 9.4, SAS Institute, Cary, NC).

The study was considered to be exempted by the University of Utah Institutional Review Board as a primary care quality improvement study.

## Results

### Chart Review

Less than 27.3% of individuals in the post-intervention sample overlapped with the pre-intervention sample. Sensitivity analyses demonstrated robustness of χ^2^ estimates for all factors, except for “other visit” (attenuated to no difference, *p* = 0.891) and being up to date on the HPV vaccine at the beginning of the visit (difference observed, *p* = 0.017), indicating the use of the full post-intervention dataset to be appropriate (Supplementary Table S1). The age distributions varied between pre- and post-intervention (Table 1). The median age increased from 17 to 18 years (*p* = 0.05), and normality assumptions appeared to be reasonable in both the pre- and post-intervention populations (X = 17.47, M = 17, s = 4.72, skew = 0.50, and kurtosis = −0.96; X = 18.99, M = 18, s = 4.61, skew = 0.09, and kurtosis = −1.37, respectively). Higher proportions of younger (ages 10–14) patients were seen prior to the intervention (34.3 vs. 21.2%), and higher proportions of older (ages 19–28) patients were seen after the intervention (49.5 vs. 33.6%). Having any HPV vaccine records on file at the time of visit differed from pre- to post-intervention (22.4 and 43.4%, respectively, *p* < 0.0001). Those without a vaccine record on file and were also not categorized as up-to-date, had an “unknown” HPV vaccination status (pre-intervention *n* = 90, 38.1% of those not up-to-date; post-intervention *n* = 43, 68.3% of those not up-to-date). Neither the number of wellness visits, patients up-to- date on an HPV vaccination schedule, patients receiving an HPV vaccination during the visit, nor patients declining a vaccination at the time of visit varied between pre- and post-intervention. A lower proportion of patients following the intervention were due for the HPV vaccination initial dose and/or booster compared with those due prior to the intervention (20.2% vs. 35.6%, *p* = 0.0035). Missed opportunities for HPV vaccination declined significantly from the pre-intervention to the post-intervention (Table 1) (21.6 vs. 8.1%, respectively, *p* = 0.002).

**Table 1:** Cross sectional review of human papillomavirus (HPV) vaccination at Telluride Medical Center (TMC) pre- and post- intervention, 2019–2021^a^.

	**Pre-intervention**		**Post-intervention**		**χ^2^ *p*-value^b^**
	**(*****n*** **=** **402)**		**(*****n*** **=** **99)**		
	**N**	**(%)**	**N**	**(%)**	
**Age at time of visit^c^**					^**^0.05
10–14	138	(34.3)	21	(21.2)	
15–18	129	(32.1)	29	(29.3)	
19–28	135	(33.6)	49	(49.5)	
**Wellness visit: Annual physical or well child check**					0.1656
No	296	(73.6)	66	(66.7)	
Yes	106	(26.4)	33	(33.3)	
**Other visit: non-wellness visit (acute care or other)**					^*^0.1222
No	103	(25.6)	33	(33.3)	
Yes	299	(74.4)	66	(66.7)	
**UTD on HPV: Up to date on HPV at beginning of visit**					0.3704
No	236	(58.7)	63	(63.6)	
Yes	166	(41.3)	36	(36.4)	
**Due for HPV: at time of visit**					^***^0.0035
No	259	(64.4)	79	(79.8)	
Yes	143	(35.6)	20	(20.2)	
**Received HPV: at visit in question**					0.8533
No	363	(90.3)	90	(90.9)	
Yes	39	(9.7)	9	(9.1)	
**Missed opportunity: patient was due for vaccine but did not receive it at visit**					^***^0.0020
No	315	(78.4)	91	(91.9)	
Yes	87	(21.6)	8	(8.1)	
**Due now: based on current date, patient is due for booster or initial vaccine**					^***^ <0.0001
No	282	(70.2)	91	(91.9)	
Yes	120	(29.9)	8	(8.1)	
**Declines: patient or parent declined at time of visit**					0.6281
No	381	(94.8)	95	(96.0)	
Yes	21	(5.2)	4	(4.0)	
**No records: patient does not have any vaccine records on file^d^**					^***^ <0.0001
No	312	(77.6)	56	(56.6)	
Yes	90	(22.4)	43	(43.4)	

a *Cross-sectional retrospective chart review performed for patients (ages 10–28 years) visiting the clinic between September 01 and December 01, 2019 (pre-intervention) or between January 15 and March 15, 2021 (post-intervention). TMC is a rural (CMS RHC, FORHP, FAR level = 4, RUCA = 10.0, RUCC = 9, UIC = 12, MUA, and HPSA for primary care and mental health) Medical Center located in Colorado*.

b *P-values shown. Pearson's χ^2^ with probability calculated for categorical variables. No overlap in 95% CIs, with normal distribution assumption, of median age pre- and post-intervention was witnessed and was confirmed in sensitivity analysis. (Pre-intervention: kurtosis= −0.96, skewness= 0.50; post-intervention kurtosis = −1.37, skewness = 0.09)*.

* *Significant at α ≤ 0.1*,

** *significant at α ≤ 0.05*,

*** *significant at α ≤ 0.01*.

c *Age collected as a continuous variable (pre-intervention X = 17.47, M = 17, s = 4.72; post-intervention X = 18.99, M = 18, s =4.61)*.

d *Those without a vaccine record on file and not up-to-date, have an “unknown” HPV vaccination status (pre-intervention n=90, 38.1% of those not up-to-date; post-intervention n=43, 68.3% of those not up-to-date)*.

### Survey Following Reminder Message Campaign

Parents/caregivers of HPV vaccine eligible patients and young adult patients at TMC who responded to the survey were primarily female (*n* = 71, 91%) and equally distributed between those 18–45 years and those over 45 years old. A little more than one-third received or remembered receiving a vaccination reminder (*n* = 28, 35.4%). Of those reporting the mode of reminder, most of them reported receiving an email message vs. a text message (*n* = 22, 68.8% vs. *n* = 10, 31.3%). Just under one-third of the parents/caregivers reported getting their child the HPV vaccination (*n* = 21, 27.3%) and slightly over one-third reported getting the HPV vaccine for themselves (*n* = 27, 34.2%).

Those who scheduled or intended to schedule an appointment differed by age (*p* = 0.0086), receipt of a reminder from TMC (*p* = 0.0002), and by vaccination status for their child (*p* = 0.0074) or themselves (*p* = 0.0116) (Table 2). Parents/caregivers who were over 45 years old had 3.46 times the unadjusted odds (95% CI 1.36–9.27) of scheduling or intending to schedule a follow-up visit as compared with those patients or parents/caregivers 18–45 years old. Those receiving the TMC reminder had 6.96 times greater odds (95% CI 2.44–22.79) of scheduling a visit as compared with those who did not recall receiving a TMC vaccination reminder. The crude odds of HPV vaccine recipients scheduling, or stating that they had scheduled, a follow-up appointment was 70% *less* than those who had not received the HPV vaccine (cOR = 0.30, 95% CI 0.11–0.77) (Table 2). However, the unadjusted odds of parents and/or caregivers of children who had received the HPV vaccine stating that they had scheduled, or were intending to scheduled, a follow-up appointment was 4.93 times (95% CI 1.51–20.59) that of those whose children had not received the vaccine. Only gender and the mode of reminder (email compared to text message) did not vary by scheduling (and intent to schedule) a follow-up appointment (*p* = 0.4153; and *p* = 0.2733, respectively), although, due to cell sizes n < 5 for both covariates, estimates were considered as unreliable.

**Table 2:** Human papillomavirus (HPV) vaccination survey following reminder intervention, Telluride Medical Center (TMC) 2020^a^.

	**Scheduled an appointment for your child or self, following the message^b^**								
	**Total**		**No and not intending**		**Yes or intend to schedule**		**χ^2^ *p*-value^c^**	**Crude odds of**	
	**(*****n*** **=** **80)**		**(*****n*** **=** **33)^b^**		**(n** **=** **41)^b^**			**scheduling/intent^c^**	
**Individual Level Variables**	**N**	**(%)**	**n**	**(%)**	**n**	**(%)**		**cOR**	**(95% CI)**
**Age**							^***^0.0086		
18-45	39	(48.8)	23	(69.7)	16	(39.0)		ref	
Over 45	41	(51.3)	10	(30.3)	25	(61.0)		**3.46**	**(1.36–9.27)**
**Gender^d^**							0.4153		
Female	71	(91.0)	29	(93.6)	36	(87.8)		ref	
Male	7	(9.0)	2	(6.5)	5	(12.2)		1.78	(0.4–10.47)
**Received TMC Reminder?**							^***^0.0002		
No	51	(64.6)	28	(84.9)	17	(42.5)		ref	
Yes	28	(35.4)	5	(15.2)	23	(57.5)		**6.96**	**(2.44–22.79)**
**Mode of Reminder?^e^**							0.2733		
Email	22	(68.8)	6	(85.7)	16	(64.0)		ref	
Text message	10	(31.3)	1	(14.3)	9	(36.0)		2.50	(0.42–26.62)
**Did your child receive the HPV vaccine?**							^***^0.0074		
No or N/A	56	(72.7)	29	(90.6)	26	(63.4)		ref	
Yes	21	(27.3)	3	(9.4)	15	(36.6)		**4.93**	**(1.51–20.59)**
**Did you receive the HPV vaccine?**							^***^0.0116		
No or N/A	52	(65.8)	15	(46.9)	31	(75.6)		ref	
Yes	27	(34.2)	17	(53.1)	10	(24.4)		**0.30**	**(0.11–0.77)**

a *Online survey of parental and/or caregiver HPV vaccination and vaccination intention following a vaccination reminder intervention at a rural (CMS RHC, FORHP, FAR level = 4, RUCA = 10.0, RUCC= 9, UIC= 12, MUA, and HPSA for primary care and mental health) Medical Center in Colorado*.

b *Participants were asked if they had scheduled an appointment for their child or self, following the appointment reminder and response options included: Yes, a well-child check (n=9, 12.2%); Yes, a lab visit for vaccines only (n = 6, 8.1%); No, but I intend to (n = 26, 35.1%); or No, and I don't plan to (n = 33, 44.6%). These responses were binarized for analysis into: No, and not intending (n = 33, 44.6%); or Yes (well-child, or lab visits) or intending to schedule (n = 41, 55.4%)*.

c Pearson χ^2^ with probability calculated. ^*^Significant at α ≤ 0.1, ^**^significant at α ≤ 0.05,

*** *significant at α ≤ 0.01. Items in bold for crude odds of scheduling, or intending to schedule, an appointment following the reminder intervention indicate statistical significance (i.e., do not include the null observation) at a 95% level*.

d *Gender categories included female, male, transgender, or other. There were n = 2 respondents that either did not respond to the question, or selected transgender, or selected other, and were not included in analyses by gender*.

e *Mode of reminder, n = 48 respondents chose answer option of “none of the above,” n = 3 indicated receiving a phone call, and n = 7 indicated getting a text message reminder. “None of above” (responses only) were excluded from analysis*.

### Healthcare Team Member Survey Following Facilitation and Education Campaign

**Figure 1:**
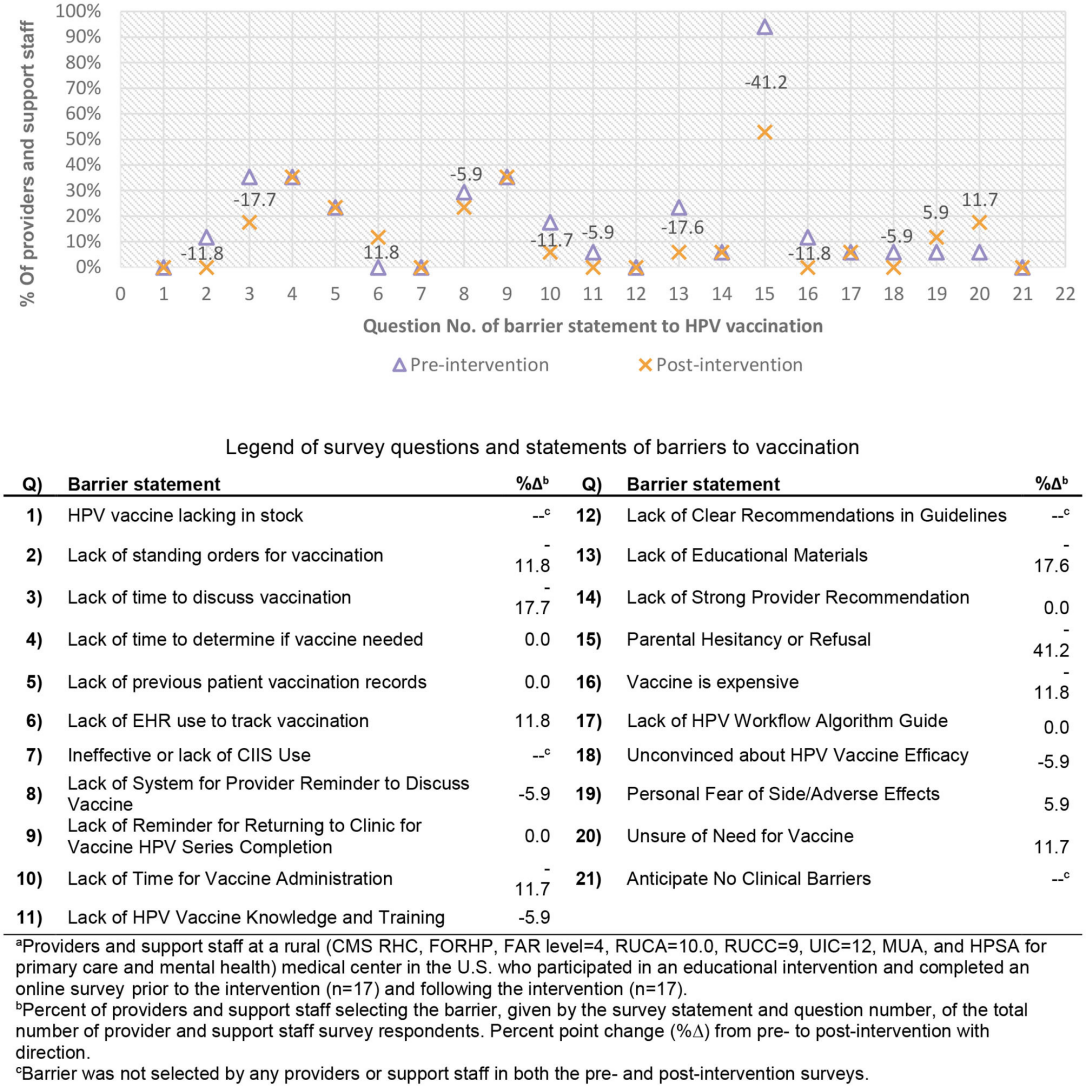
Pre- and post-intervention survey of encountered HPV vaccination barriers among providers and support staff^a^ with percent point change^b^. Legend of survey questions and statements of barriers to vaccination.

The number of providers and support staff completing the pre-intervention and post-intervention surveys did not change (*n* = 17). Survey responses were not paired from pre- to post-intervention and results represent changes at the clinic level. Most respondents agreed or strongly agreed to utilize education and resources in the future (*n* = 17, 100%, data not shown), and that the education and materials provided were helpful in treating every visit like a vaccination visit (*n* = 14, 82.4%, data not shown). The number of providers and support staff identifying a lack of educational materials as a barrier to vaccination decreased more than 17% points following the intervention, as did lack of time to discuss vaccination (Figure 1). The most frequently identified barrier to HPV vaccination selected by providers and support staff at TMC, in both the pre- and post-healthcare team training surveys was parental vaccination hesitancy or refusal, which declined 41.2% points, the greatest decrease (by percentage point) seen across vaccination barriers. Lack of standing orders (−11.8%), lack of time to administer the immunization (−11.7%), cost (vaccine is expensive) (−11.8%), lack of a provider reminder system for discussing vaccination (−5.9%), lack of HPV knowledge and training (−5.9%), and being unconvinced of the vaccine efficacy (−5.9%) all decreased by more than 5% points following the intervention. There were three (3) barriers that were selected more frequently by providers and support staff following the campaign and those included: lack of EHR use to track vaccination (11.8%), being unsure of the need for vaccination (11.7%), and a personal fear of adverse and/or side effects (5.9%), which all increased by at least 5% points (Figure 1).

When examining what had been helpful in facilitating HPV vaccination by providers and support staff *via* free-text responses (data not shown), three general themes emerged as helpful: framing HPV vaccination as cancer prevention, education and materials provided, and education and discussion on HPV and vaccinating both male and female patients prior to the 15th birthday of the patient. An example of a statement of framing HPV vaccination as cancer prevention was “We have really taken on the approach as presenting this vaccine as part of cancer prevention, which parents are much more likely to receive well”. A characterization of the helpfulness of the education and discussion of HPV and vaccination was “We also have been able to provide valuable statistics on the prevalence of HPV once a patient becomes sexually active, and by stressing that we vaccinate early when children can mount a strong response long before they are exposed has helped parents understand why we vaccinate early against HPV”. Feasibility measures and usability among providers and support staff at TMC were favorable overall in both the pre- and post-intervention surveys (Table 3), with most strongly agreeing that: “…the benefits of current practices in place for HPV vaccination at my facility outweigh the costs/risks” (70.6 and 52.9%, respectively), “…the practices in my clinical setting are valuable and address barriers to HPV vaccination” (47.1 and 64.7%, respectively), and “current strategies I use for HPV vaccination in my clinic are easy to understand” (58.8 and 72.7%, respectively). Although, none of the feasibility and usability measures showed a significant difference (Table 3) following the facilitation and education campaign intervention (*p* > 0.2 for each).

**Table 3:** Survey of feasibility and usability of human papillomavirus (HPV) vaccination practices according to providers and support staff at a rural Medical Center following an educational campaign, 2020–2021.

	**Pre-intervention**		**Post-intervention**		**χ^**2**^ *p*-value^a^**
	***n***	**(%)**	***n***	**(%)**	
**Feasibility Measure**					
*I believe the benefits of current practices in place for HPV vaccination at my facility outweigh the costs/risks*					0.2897
Strongly agree	12	(70.6)	9	(52.9)	
Agree; Neither agree nor disagree	5	(29.4)	8	(47.1)	
**Usability Measures**					
*I believe practices in my clinical setting are valuable and address barriers to HPV vaccination*					0.3001
Strongly agree	8	(47.1)	11	(64.7)	
Agree; Disagree	9	(52.9)	6	(35.3)	
*Current strategies I use for HPV vaccination in my clinic are easy to understand*					0.4533
Strongly agree	10	(58.8)	8	(72.7)	
Agree; Neither agree nor disagree; Strongly disagree	7	(41.2)	3	(27.3)	

a *Pearson's χ^2^, P (df = 1) reported*.

## Discussion

Overall, the multi-level and multi-mode HPV vaccination intervention performed well in a rural setting, even during the COVID-19 pandemic. Our findings indicate that patients who did not receive the HPV vaccine at the time of visit, although they were due for the vaccination (i.e., missed opportunities), declined significantly following the HPV vaccination intervention period. Parental vaccination hesitancy or refusal, the most commonly reported vaccination barrier by providers and support staff, also declined by 41.2% points.

While most barriers decreased in the frequency of selection by providers and support staff, there were a few vaccination barriers that were selected more frequently following the campaign. These included: lack of EHR use to track vaccination; personal fear of adverse and/or side effects; and being unsure of the need for vaccination. The lack of a system for provider reminders to discuss immunization decreased during a time of clinic overburden from COVID-19, while lack of an EHR to track vaccination and being unsure of the need for vaccination both increased following the education intervention. This likely characterizes sentiments expressed by providers and support staff in person during the educational campaign of a lack of a national immunization registry and a largely seasonal and/or transient patient-population that is often unsure of their own and/or child's HPV vaccination status. Future work for enhancing immunization uptake, particularly in rural settings, could include better integration of immunization registries at a national level with enhanced optimization of provider reminders which would improve provider and support staff confidence for the need of vaccination among transitory patient populations.

These early results are promising but without a control group, or comparison to another similar clinic during the same time period, we cannot characterize the effect or the degree of effect the intervention had vs. other external factors. Future analyses could include difference-in-difference calculations to extend these findings from association into causal. Small cell sizes, due largely to unequal pre-post intervention population sizes, were present. At this time, we are unable to accurately characterize the impact of the COVID-19 pandemic on HPV vaccination in this population. The COVID-19 pandemic paused regular in-person appointments for many and may also have resulted in fewer people getting the HPV vaccine; counts may be underestimated as some people may get vaccinated once the pandemic is over; or counts may be accurate as some people may forego vaccination. This would be consistent with previous research indicating a preference for a single vaccine per visit, and many may prioritize the COVID-19 vaccine over the HPV vaccine.

The results of this study suggest that, among rural patients aged 18 and older, those receiving a clinic HPV vaccination reminder, regardless of the mode, are 6.96 times more likely to schedule or intend to schedule a follow-up visit for vaccination for either their child or themselves (cOR = 6.96, 95% CI 2.44–22.79). Small numbers (n < 5) in several of the cells in the contingency table of scheduling a follow-up visit contributed to imprecise (i.e., wide *CIs*) and unstable estimates of probability and odds for gender, mode of reminder, and a child's receipt of the HPV vaccine. It is unclear why so many people reported not receiving the reminder messages. This could be due to limited patient recall, or inaccurate records of patient contact information or contact preferences. Future studies could examine this issue in an attempt to increase acceptance, receipt, and/or memorability of messages.

The population of providers and support staff, while sizable for a single-clinic rural intervention, suffered from small cell sizes issues and some unstable estimates were present. Additionally, the responses were unpaired and we were unable to measure individual changes in responses or knowledge improvement from prior to and following the facilitation and education campaign. None of the comparisons of pre- to post-intervention survey responses from providers and support staff were statistically significant at α = 0.05, however, many of the outcomes demonstrated improvement and have important clinical implications for HPV administration and championing vaccination for the future. Caution should be taken in generalizing our results to all rural areas and the unique regional, cultural, economic, and medical landscape should be considered before implementing any public health campaign among vulnerable populations. We are unsure how the pandemic will influence HPV vaccination over time.

Results from this multi-level and multi-model intervention are promising and suggest that vaccination interventions consisting of provider and support staff education and training activities, parent/caregiver and young adult reminders, and evidence-based patient education materials can reduce missed opportunities for HPV vaccination in a rural setting. Future research should assess the implementation of larger scale multi-level and multi-model HPV vaccination interventions in rural primary care settings across the United States to reduce inequities in HPV vaccination rates and the incidence and mortality of HPV-related cancers among these vulnerable patient populations.

## Data Availability Statement

The raw data supporting the conclusions of this article will be made available by the authors, without undue reservation.

## Ethics Statement

The studies involving human participants were reviewed and approved by University of Utah Institutional Review Board. Written informed consent for participation was not required for this study in accordance with the national legislation and the institutional requirements.

## Author Contributions

DK designed the study, led the data collection and analysis activities, and interpreted the results. EM and AW designed the HPV vaccine training and intervention activities. GF and BG are our clinical informatics experts and contributed to the design of the digital intervention. SA, HB, SM, and AP assisted with interpretation of findings. GC provided senior input on study design and interpretation of findings. DK, KC, and SA drafted the manuscript. All authors reviewed and provided edits to the manuscript.

## Conflict of Interest

DK receives a small portion of her salary from a grant that is provided and supported by the American Cancer Society, who received funding from Merck, for the purpose of the “Mission: HPV Cancer Free Quality Improvement Initiative.” EM receives a small portion of her salary from a memorandum of understanding with the Huntsman Cancer Institute. The remaining authors declare that the research was conducted in the absence of any commercial or financial relationships that could be construed as a potential conflict of interest.

## Publisher's Note

All claims expressed in this article are solely those of the authors and do not necessarily represent those of their affiliated organizations, or those of the publisher, the editors and the reviewers. Any product that may be evaluated in this article, or claim that may be made by its manufacturer, is not guaranteed or endorsed by the publisher.
