# Supplementary material for: Successful Multi-Level HPV Vaccination Intervention at a Rural Healthcare Center in the Era of COVID-19
Source: Front Digit Health. 2021 Aug 19;3:719138. doi: 10.3389/fdgth.2021.719138 (PMC8521914; doi:10.3389/fdgth.2021.719138)
Supplement: Supplementary file 1 [file Table_1.pdf]

Table S1. Sensitivity analysis for cross sectional review of HPV vaccination at Telluride Medical Center (TMC) pre- and post-intervention, 2019-2021[a]

	Pre-Intervention (n=402)		Post-Intervention (n=99)		X^2	Post-Intervention, Overlaps Removed (n=72)		X^2
	n	(%)	n	(%)	*p-value*[b]	n	(%)	*p-value*[b]
**Age at time of visit**[c]					**0.05			**0.05
10-14	138	(34.3)	21	(21.2)		13	(18.1)	
15-18	129	(32.1)	29	(29.3)		15	(20.8)	
19-28	135	(33.6)	49	(49.5)		44	(61.1)	
**Wellness visit: Annual physical or well child check**					0.1656			0.9971
No	296	(73.6)	66	(66.7)		53	(73.6)	
Yes	106	(26.4)	33	(33.3)		19	(26.4)	
**Other visit: non-wellness visit (acute care or other)**					*0.1222			0.891
No	103	(25.6)	33	(33.3)		19	(26.4)	
Yes	299	(74.4)	66	(66.7)		53	(73.6)	
**UTD on HPV: Up to date on HPV at beginning of visit**					0.3704			**0.017
No	236	(58.7)	63	(63.6)		53	(73.6)	
Yes	166	(41.3)	36	(36.4)		19	(26.4)	
**Due for HPV: at time of visit**					***0.0035			***0.0003
No	259	(64.4)	79	(79.8)		62	(86.1)	
Yes	143	(35.6)	20	(20.2)		10	(13.9)	
**Received HPV: at visit in question**					0.8533			0.4578
No	363	(90.3)	90	(90.9)		67	(93.1)	
Yes	39	(9.7)	9	(9.1)		5	(6.9)	
**Missed opportunity: patient was due for vaccine but did not receive it at visit**					***0.0020			***0.0014
No	315	(78.4)	91	(91.9)		68	(94.4)	
Yes	87	(21.6)	8	(8.1)		4	(5.6)	
**Due now: based on current date, patient is due for booster or initial vaccine**					***<.0001			***<.0001
No	282	(70.2)	91	(91.9)		68	(94.4)	
Yes	120	(29.9)	8	(8.1)		4	(5.6)	
**Declines: patient or parent declined at time of visit**					0.6281			0.3737
No	381	(94.8)	95	(96.0)		70	(97.2)	
Yes	21	(5.2)	4	(4.0)		2	(2.8)	
**No records: patient does not have any vaccine records on file**					***<.0001			***<.0001
No	312	(77.6)	56	(56.6)		29	(40.3)	
Yes	90	(22.4)	43	(43.4)		43	(59.7)	

[a]Cross sectional retrospective chart review performed for patients (ages 10-28 years) visiting the clinic between 09/01/2019 and 12/01/2019 (pre-intervention) or between 01/15/2021 and 03/15/2021 (post-intervention). TMC is a rural (CMS RHC, FORHP, FAR level=4, RUCA=10.0, RUCC=9, UIC=12, MUA, and HPSA for primary care and mental health) medical center located in Colorado.

[b]P-values shown. Pearson χ^2 with probability calculated for categorical variables. No overlap in 95% CIs, with normal distribution assumption, of median age pre- and post-intervention was witnessed and was confirmed in sensitivity analysis. (Pre- intervention: kurtosis= -0.96, skewness= 0.50; Post-intervention kurtosis= -1.37, skewness= 0.09; Limited post-intervention: kurtosis= -1.28, skewness= -0.30). *Significant at $\alpha \leq 0.1$, **significant at $\alpha \leq 0.05$, ***significant at $\alpha \leq 0.01$

[c]Age collected as a continuous variable (Pre-intervention $\bar{X}$=17.47, M=17, s=4.72; Post-intervention $\bar{X}$=18.99, M=18, s=4.61; Limited post-intervention: $\bar{X}$=19.85, M=21, s=4.73).

[d]Those without a vaccine record on file and not up-to-date, have an "unknown" HPV vaccination status (pre-intervention n=90, 38.1% of those not up-to-date; post-intervention n=43, 68.3% of those not up-to-date).
